# Supplementary material for: Scrutinizing GW-Based Methods Using the Hubbard Dimer
Source: Front Chem. 2021 Oct 29;9:751054. doi: 10.3389/fchem.2021.751054 (PMC8586429; doi:10.3389/fchem.2021.751054)
Supplement: Supplementary file 1 [file DataSheet1.pdf]

Supplementary Material

1 G_0W_0 EQUATIONS FOR THE HALF-FILLED HUBBARD DIMER

Starting from G_0, which reads

$$G_{0,IJ}(\omega) = \frac{1}{2}\left[\frac{1}{\omega + t - \mathrm{i}\eta} + \frac{(-1)^{(I-J)}}{\omega - t + \mathrm{i}\eta}\right], \tag{S1}$$

one obtains the same P and W given in Eqs (21)-(22) (of the main article), from which the G_0W_0 self-energy reads

$$\Sigma_{IJ}(\omega) = \frac{U}{2}\delta_{IJ} + \frac{U^2 t}{2h}\left[\frac{1}{\omega - (t+h) + \mathrm{i}\eta} + \frac{(-1)^{I-J}}{\omega + (t+h) - \mathrm{i}\eta}\right]. \tag{S2}$$

One then obtains the following G_0W_0 removal/addition energies

$$\epsilon_{1,\pm} = +\frac{h}{2} + \frac{U}{4} \pm \frac{\sqrt{(h + 2t - U/2)^2 + 4tU^2/h}}{2}, \tag{S3a}$$

$$\epsilon_{2,\pm} = -\frac{h}{2} + \frac{U}{4} \pm \frac{\sqrt{(h + 2t + U/2)^2 + 4tU^2/h}}{2}, \tag{S3b}$$

with the quasiparticle solutions being $\epsilon^{\mathrm{QP}}_{\mathrm{bn}} = \epsilon_{1,-}$ and $\epsilon^{\mathrm{QP}}_{\mathrm{an}} = \epsilon_{2,+}$, which correspond to the bonding and antibonding energies, respectively. The corresponding renormalization factors read

$$Z^{\mathrm{QP}}_{\mathrm{bn}} = \frac{1}{2} + \frac{h + 2t - \frac{U}{2}}{2\sqrt{(h + 2t - U/2)^2 + 4tU^2/h}}, \tag{S4a}$$

$$Z^{\mathrm{QP}}_{\mathrm{an}} = \frac{1}{2} + \frac{h + 2t + \frac{U}{2}}{2\sqrt{(h + 2t + U/2)^2 + 4tU^2/h}}, \tag{S4b}$$

and $Z^{\mathrm{sat}}_{\mathrm{bn/an}} = 1 - Z^{\mathrm{QP}}_{\mathrm{bn/an}}$. We notice that the removal/addition energies and corresponding intensities given in Eqs (S3a)-(S4b) correspond to the expressions (35) and (43)-(44) given in Ref. (Hellgren et al., 2015) upon setting the nearest neighbour interaction U_1 to zero and $\epsilon_{H/L} = \pm t$.

2 NUMERICAL EVGW AND SCGW CALCULATIONS

Following Ref. (Puig von Friesen et al., 2010), we see from Eq. (1) (of the main article) that the matrix elements of the exact Green's function G in a generic orbital basis set can be expressed in the frequency domain as a sum over poles, *i.e.*,

$$G_{ij}(\omega) = \sum_{\nu} \frac{G_{ij,\nu}}{\omega - \epsilon_\nu + \mathrm{i}\eta\,\mathrm{sgn}(\epsilon_\nu - \mu)}, \tag{S5}$$

where we introduced the spectral intensities

$$G_{ij,\nu} = \int d\boldsymbol{x}_1\boldsymbol{x}_2\phi_i^*(\boldsymbol{x}_1)\psi_\nu(\boldsymbol{x}_1)\psi_\nu^*(\boldsymbol{x}_2)\phi_j(\boldsymbol{x}_2). \tag{S6}$$

This representation remain valid for approximate Green's function, such as the non-interacting G or its mean-field versions. Likewise, Σ, W, and P have similar representations. Equation (S5) allows us to evaluate convolutions and cross-correlations analytically. Given the two functions

$$A(\omega) = \sum_\nu \frac{A_\nu}{\omega - a_\nu + \mathrm{i}\eta\,\mathrm{sgn}(a_\nu - \mu)}, \tag{S7a}$$

$$B(\omega) = \sum_\nu \frac{B_\nu}{\omega - b_\nu + \mathrm{i}\eta\,\mathrm{sgn}(b_\nu - \mu)}, \tag{S7b}$$

their cross correlation functions

$$C(\omega) = \int \frac{d\omega'}{2\pi\mathrm{i}} A(\omega')B(\omega + \omega'), \tag{S8a}$$

$$D(\omega) = \int \frac{d\omega'}{2\pi\mathrm{i}} A(\omega')B(\omega - \omega'), \tag{S8b}$$

can be written as

$$\begin{aligned} C(\omega) = &- \sum_{b_\nu<\mu}\sum_{a_\xi>\mu} \frac{A_\xi B_\nu}{\omega - (b_\nu - a_\xi) - \mathrm{i}\eta} \\ &+ \sum_{a_\nu<\mu}\sum_{b_\xi>\mu} \frac{A_\nu B_\xi}{\omega - (b_\xi - a_\nu) + \mathrm{i}\eta}, \end{aligned} \tag{S9}$$

$$\begin{aligned} D(\omega) = &\sum_{a_\nu<\mu}\sum_{b_\xi<\mu} \frac{A_\nu B_\xi}{\omega - (a_\nu + b_\xi) - \mathrm{i}\eta} \\ &- \sum_{a_\nu>\mu}\sum_{b_\xi>\mu} \frac{A_\nu B_\xi}{\omega - (a_\nu + b_\xi) + \mathrm{i}\eta}. \end{aligned} \tag{S10}$$

Equations (S8a) and (S8b) enter, for example, in the evaluation of P and Σ.

The Dyson equation for G can then be solved in two steps: i) finding the poles of G, $\epsilon_{i,\nu}^{GW}$, which correspond to the zeroes of Eq. (5) (of the article) with $\phi_i(\boldsymbol{r}) = \phi_{\mathrm{bn/an}}(\boldsymbol{r})$, with, for example, an ordinary root finding algorithm; ii) once the positions of the poles are known, one can then compute the corresponding spectral weights via Eq. (6) (of the article).

REFERENCES

Hellgren, M., Caruso, F., Rohr, D. R., Ren, X., Rubio, A., Scheffler, M., et al. (2015). Static correlation and electron localization in molecular dimers from the self-consistent RPA and G W approximation. Phys. Rev. B 91, 165110. doi:10.1103/PhysRevB.91.165110

Puig von Friesen, M., Verdozzi, C., and Almbladh, C.-O. (2010). Kadanoff-baym dynamics of hubbard clusters: Performance of many-body schemes, correlation-induced damping and multiple steady and

quasi-steady states. Phys. Rev. B 82, 155108. doi:10.1103/PhysRevB.82.155108
